# Rare *Enterobius vermicularis* infection of the greater omentum misdiagnosed as schistosomiasis: a case report

**DOI:** 10.1186/s40249-026-01429-6

**Published:** 2026-03-03

**Authors:** Yumeng Cai, Feng Miao, Muxin Chen, Jiahui Sun, Yahong Hu, Xiaoyu Qin, Yu Luo, Haiting Xiao, Yuchun Cai, Xiaonong Zhou

**Affiliations:** 1 National Key Laboratory of Intelligent Tracking and Forecasting for Infectious Diseases, National Institute of Parasitic Diseases, Chinese Center for Disease Control and Prevention (Chinese Center for Tropical Diseases Research), Shanghai, 200025 China; 2 National Institute of Parasitic Diseases, Chinese Center for Disease Control and Prevention (Chinese Center for Tropical Diseases Research), Laboratory of Parasite and Vector Biology, Ministry of Public Health, WHO Collaborating Centre for Tropical Diseases, National Center for International Research on Tropical Diseases, Ministry of Science and Technology, Shanghai, 200025 China; 3 Shandong Institute of Parasitic Diseases, Shandong First Medical University & Shandong Academy of Medical Sciences, Jining, China

**Keywords:** *Enterobius vermicularis*, Ectopic enterobiasis, Misdiagnosis, Schistosomiasis, Greater omentum

## Abstract

**Background:**

Enterobiasis, caused by *Enterobius vermicularis,* is a common intestinal parasitic infection in children. Ectopic migration to extraintestinal sites, such as the greater omentum, is rare and often misdiagnosed due to nonspecific clinical manifestations and limited proficiency in identifying parasitic structures in paraffin-embedded histological sections.

**Case presentation:**

A 12-year-old female presented with a three-day history of lower abdominal pain and a pelvic mass. Emergency laparoscopic resection revealed an ovarian serous cystadenoma and an omental mass. Initial histopathological examination of the omental mass suggested schistosome eggs; however, expert consultation confirmed a section of an adult female *E. vermicularis* containing eggs measuring up to 50 μm. The patient had no exposure to schistosomiasis-endemic areas but a history of prior pinworm infection, which had been treated with oral albendazole (400 mg once daily for 2 days). Subsequent adhesive tape tests over three consecutive days were negative, and perineal pruritus was resolved, confirming successful cure. The final diagnosis was ectopic enterobiasis of the greater omentum.

**Conclusions:**

This case underscores the critical role of accurate morphological identification in distinguishing parasitic infections. Misdiagnosis, even in non-schistosomiasis-endemic areas, reflects insufficient training in parasitic morphology among healthcare professionals. Enhanced training on the morphology of common parasites and interpretation of paraffin-embedded histological sections is essential to improve diagnostic accuracy.

**Graphical Abstract:**

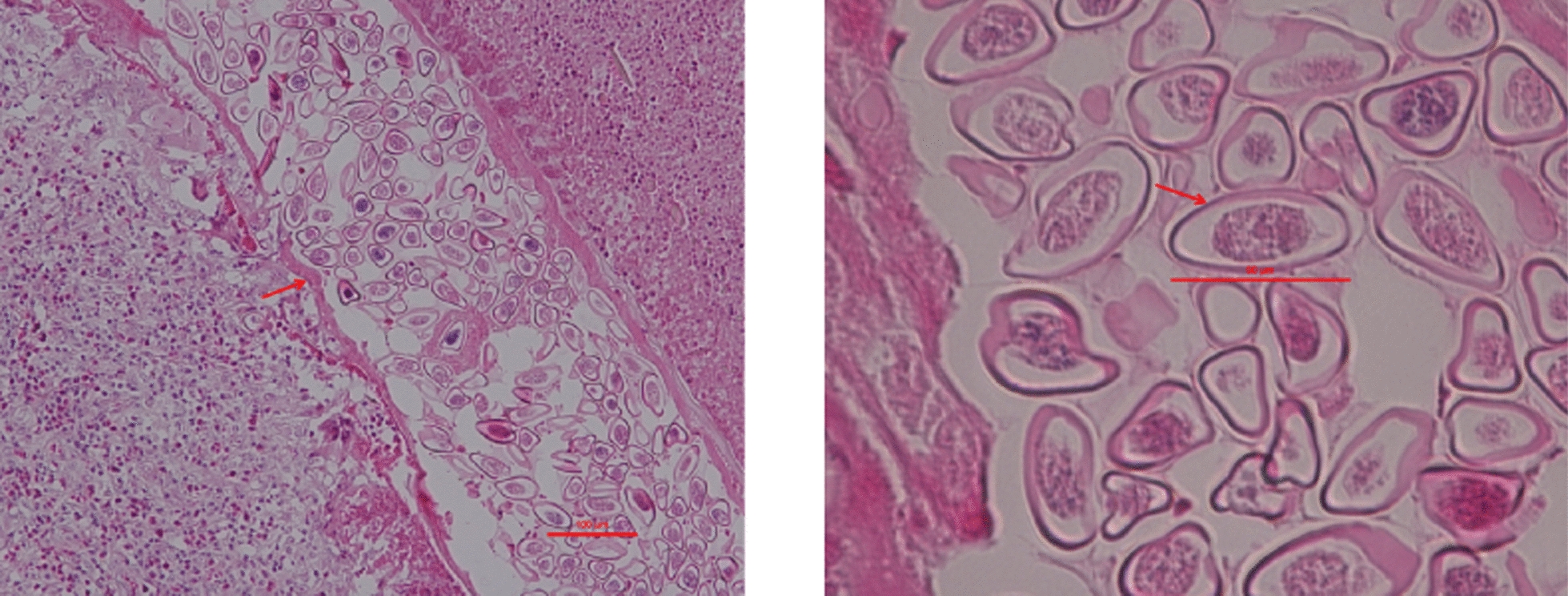

**Supplementary Information:**

The online version contains supplementary material available at 10.1186/s40249-026-01429-6.

## Background

Enterobiasis is a common parasitic disease caused by the intestinal nematode *Enterobius vermicularis,* which primarily infects the human gastrointestinal tract and is particularly prevalent among children [1–4]. It is recognized worldwide as a neglected parasitic disease [5]. Epidemiological data indicate that, in certain regions of China, the prevalence of *E. vermicularis* among children aged 3–12 years reaches 1.37% [6]. The primary clinical manifestation of enterobiasis is perianal and perineal pruritus, which results from female worms migrating to the anal region to deposit eggs at night [1, 2]. Eosinophilia may also serve as an important indicator of pinworm infection, especially in cases of ectopic parasitism [7].

Although *E. vermicularis* typically inhabits the large intestine, ectopic migration to extraintestinal sites, though rare, has been reported [4, 8, 9]. Ectopic parasitism refers to the invasion of tissues or migration of adult worms to sites not normally inhabited by the parasite. Such infections have been documented in multiple sites, including the female genital tract, abdominal cavity, pelvis, appendix, liver, kidneys, and prostate [4, 6, 8–15]. Clinically, ectopic infections often present with nonspecific symptoms, such as abdominal pain, abnormal bleeding, or masses. They may induce inflammatory granulomas [16] or pseudotumorous lesions[17], which can mimic malignancies or tuberculosis [9, 16], potentially leading to misdiagnosis and unnecessary surgical intervention [10, 17].

A key diagnostic challenge in ectopic enterobiasis is the limited sensitivity and ineffectiveness of conventional stool examinations and adhesive tape tests. Consequently, diagnosis often relies on postoperative pathological confirmation [18]. Accurate morphological identification of the parasite is therefore essential for definitive diagnosis [5, 8, 9]. Notably, with major progress in parasitic disease control across China, both endemic areas and infection rates have dropped significantly. As a result, healthcare professionals now have far fewer opportunities to encounter such cases in clinical practice, which has gradually eroded their familiarity with common parasitic pathogens [5, 9]. This knowledge gap is particularly prominent in non-endemic regions, where parasitic infections may be misdiagnosed due to limited expertise in identifying parasitic structures in paraffin-embedded tissue sections. Given the rarity of ectopic enterobiasis involving the greater omentum and the high risk of misdiagnosis as other diseases, such as schistosomiasis, this case report describes a clinical case of ectopic *E. vermicularis* infection in the greater omentum. It further highlights the importance of accurate morphological identification in conjunction with a clinical-epidemiological context, thereby providing a valuable reference to improve diagnostic accuracy in clinical practice.

## Case presentation

### Patient history and admission findings

A 12-year-old female from Juye County, Heze City, Shandong Province, China, was admitted on July 20, 2019, with a 3-day history of lower abdominal pain. Her medical history was notable for vitiligo of more than one year’s duration and a prior diagnosis of pinworm infection one year earlier. At that time, she presented with anal itching, and pinworm eggs were detected using the perianal adhesive tape test conducted once daily for three consecutive mornings prior to perianal washing or hygiene measures. She was treated with oral albendazole (400 mg once daily for 2 days). Three follow-up perianal adhesive tape tests, following the same protocol, yielded negative results and her anal itching was resolved, confirming successful treatment. She denied any history of hepatitis, tuberculosis, or other infectious diseases. She had not yet experienced menarche, was unmarried and nulliparous. She was born and had continuously resided in her hometown, with no history of prolonged residence elsewhere or travel to schistosomiasis-endemic areas.

On admission, the patient was conscious and in fair spirits, with normal diet, sleep, and bowel habits, and stable weight. Vital signs were as follows: temperature 36.7 °C, pulse 104 beats/min, respiration 25 breaths/min, and blood pressure of 83/54 mmHg. She was a female child with normal development and moderate nutritional status, who was conscious and cooperative during the examination. Multiple white patchy skin lesions consistent with vitiligo were observed. No rash, hemorrhagic spots, jaundice, spider nevi, or superficial lymphadenopathy were noted.

### Surgical intervention

On July 20, 2019, the patient underwent an emergency laparoscopic ovarian tumor debulking (right) combined with omental mass resection. The right ovarian serous cystadenoma was completely excised. Post-operative recovery was uneventful, with no evidence of residual or recurrent disease. She was discharged on July 24, 2019, with well-healed surgical wounds and was transferred on the same day to the Shandong Institute of Parasitic Disease Control for further evaluation and treatment of a suspected schistosome egg structure in the omental tissue.

## Diagnosis

### Imaging and pathological examinations

**Fig. 1:**
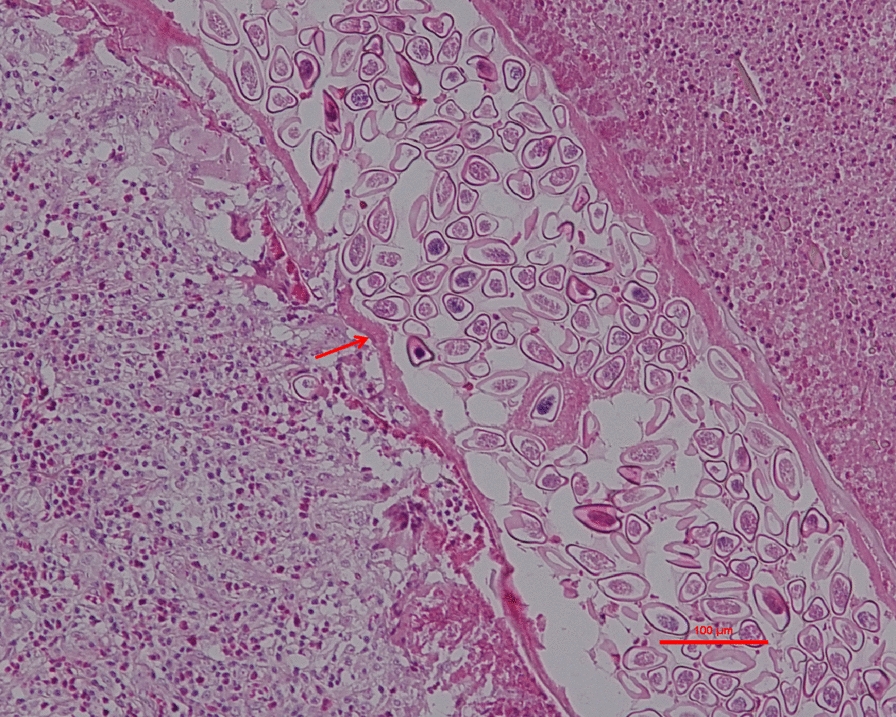
Pathological section of the greater omentum from a 12-year-old female patient with ectopic enterobiasis. Hematoxylin and eosin staining show a section of an adult female *Enterobius vermicularis*, with visible characteristic structures including the cuticle, muscle layer, and uterine structure, features critical for confirming parasitic infestation. Scale bar size: 100 μm

**Fig. 2:**
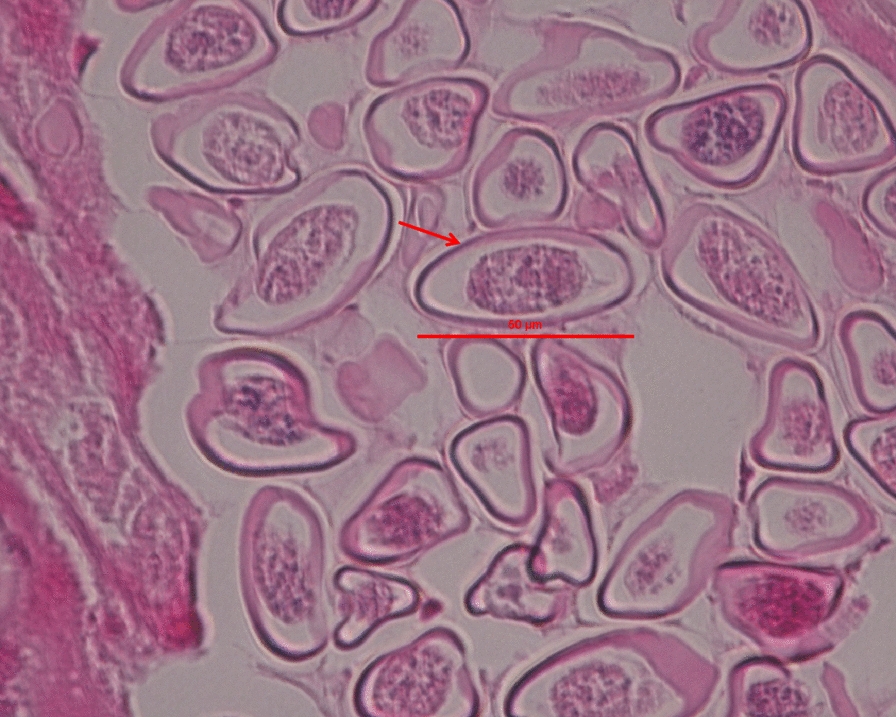
Histopathological features of *Enterobius vermicularis* eggs in the greater omentum of a 12-year-old female with ectopic infection. Hematoxylin and eosin staining display multiple *E. vermicularis* eggs within the uterus of the adult female worm, observed in transverse, oblique, and longitudinal sections. The eggs are asymmetrically oval, with a transparent eggshell containing an embryo and measure up to 50 μm in length. Scale bar size: 50 μm

Gynecological ultrasound indicated a pelvic mass measuring approximately 10.9 cm × 8.5 cm × 5.5 cm. Postoperative pathological examination confirmed a right ovarian serous cystadenoma. Subsequent microscopic evaluation of the omental tissue at the Shandong Institute of Parasitic Disease Control, following hematoxylin and eosin staining, showed fibrous connective and adipose tissues with lymphoid hyperplasia, extensive eosinophilic infiltration, and areas of necrosis. Initial histopathological examination of the omental mass suggested the presence of schistosome eggs (Supplementary Figures S1, S2). However, subsequent expert consultation confirmed that the specimen represented a cross-section of an adult female *Enterobius vermicularis*, with identifiable morphological features including the cuticle, muscle layer, and internal structures (e.g., uterus) (Fig. 1). Numerous pinworm eggs were observed within the uterus in transverse, oblique, and longitudinal sections, with a maximum length of approximately 50 μm (Fig. 2).

### Diagnostic basis

The final diagnosis was ectopic *E. vermicularis* infection of the greater omentum. The diagnosis was based on the patient’s clinical presentation, including lower abdominal pain and a surgically resected pelvic mass. Histopathological examination of the greater omentum revealed key inflammatory changes and identifiable parasitic structures. Consultation with a specialized parasitic disease institution confirmed that the microscopic findings were consistent with a cross-section of an adult female pinworm. Characteristic structures, including the cuticle, muscle layer, and uterus, were clearly identified. Numerous typical pinworm eggs were observed within the uterus, presenting as an asymmetrically oval shape with a transparent eggshell and a visible embryo. The patient resided in Juye County, Heze City, Shandong Province, where the reported prevalence of *E. vermicularis* among children aged 3–12 years is 1.37% [6]. The region is non-endemic for schistosomiasis, and the patient had no history of travel to schistosomiasis-endemic regions, thereby excluding schistosome infection. Furthermore, the absence of current perineal pruritus and negative adhesive tape tests during the current admission supported the absence of active intestinal pinworm infection.

No additional anti*-Enterobius* therapy was administered, as the findings did not indicate an active infection. The patient was followed up via telephone for 6 months after the final diagnosis. During this period, no recurrence of abdominal pain, pelvic masses, or anal itching was reported, and her overall health remained stable.

## Discussion

This 12-year-old pre-menarche female developed an inflammatory mass in the greater omentum due to ectopic *E. vermicularis* parasitism, characterized by extensive eosinophilic infiltration, lymphoid hyperplasia, and necrosis. The proposed mechanism involves upward migration of female pinworms through the vulva and vagina after oviposition in the anal folds, with subsequent colonization of the greater omentum. A prior pinworm infection was considered a key predisposing factor. Notably, the initial misdiagnosis of schistosome eggs was attributable to two critical issues. First, healthcare providers in non-endemic regions lacked familiarity with the morphological characteristics of schistosome eggs, which vary by species. These eggs are typically oval, pale yellow, and thin-shelled, with species-specific spine positioning. However, the spine is often inconspicuous in histological sections, especially for *Schistosoma japonicum*. The eggs may also contain a miracidium with typical internal structures. Second, insufficient integration of the clinical-epidemiological context contributed to the diagnostic error. Schistosome eggs typically measure 70–100 μm × 50–65 μm, which is clearly larger than the pinworm eggs observed in this patient (maximum length approximately 50 µm). Moreover, the patient had no history of travel to schistosomiasis-endemic areas, a key epidemiological detail that should have excluded schistosomiasis. This case underscores the need for effective communication between clinicians and pathologists.

In patients presenting with abdominal CT findings of hazy omentum or mass lesions, parasitic infections, including pinworms, pulmonary flukes, and schistosomiasis, should be considered in the diagnosis [12, 19–22]. Recent studies suggest that ectopic migration may occur when female pinworms accidentally enter adjacent cavities during oviposition or through hematogenous dissemination [4, 13, 23–25]. Table 1 summarizes the diverse clinical manifestations and diagnostic challenges associated with ectopic *E. vermicularis* infections to aid clinician recognition.

**Table 1:** Clinical features and diagnostic challenges of ectopic *Enterobius vermicularis* infections

Site of ectopic infection	Clinical manifestations/lesions	Common misdiagnoses	Key diagnostic challenges	References
Female genital tract	Inflammation, mass formation, abnormal bleeding	Malignancies, pelvic tumors	Insensitive stool/cellophane tape tests, often diagnosed postoperatively	[4, 8–10, 26]
Abdominal cavity	Granulomatous inflammation, pseudotumorous lesions, abdominal pain	Tuberculosis, malignancies	Relies on pathological confirmation after surgery or biopsy	[10, 11, 18]
Appendix	Acute appendicitis-like symptoms, inflammation	Acute appendicitis, other abdominal emergencies	Difficult to distinguish from common appendicitis preoperatively	[12, 23]
Liver	Granulomatous inflammation, pseudotumorous lesions, abdominal pain, hepatomegaly	Hepatic tumors, tuberculosis, liver abscess	Extremely low clinical suspicion due to rarity, conventional stool exams are ineffective, definitive diagnosis relies on postoperative pathological identification of adult worms or eggs within granulomas	[13]
Kidneys	Granulomatous inflammation, mass formation, hematuria, and flank pain	Renal tumor, tuberculosis, other granulomatous diseases	Non-specific symptoms, low clinical suspicion, definitive diagnosis relies on pathological examination of biopsy or surgical specimen	[14]
Prostate	Prostatitis-like symptoms, granulomatous inflammation, pseudotumorous lesions	Chronic prostatitis, prostate cancer, tuberculosis	Non-specific symptoms overlapping with common prostate conditions, requires histopathological confirmation via biopsy	[15]

Healthcare personnel at all levels require systematic training in parasitic morphology and the interpretation of histopathological sections. This dual deficiency in knowledge and skills is concerning, given the frequent clinical detection of pinworm eggs and the potential for inappropriate management resulting from misidentification. In managing pinworm infections, the familial aggregation of infection, in addition to synchronized pharmacological treatment of all household members to prevent cross-infection, supplementary control measures should be implemented based on established infection control principles. All beddings, towels, linens, and stuffed animals should be washed on the day of treatment. In daycare or preschool settings, treatment of close contacts should be considered if applicable. Patients should shower or wash the anal region in the morning to remove eggs deposited overnight and minimize environmental contamination. A second course of treatment is recommended 2 weeks after the initial dose to eliminate developing pre-adult worms and prevent reinfection from residual environmental eggs.

## Conclusions

This report describes a 12-year-old pre-menarche female who developed an inflammatory greater omental mass caused by ectopic *E. vermicularis* infection. The condition was likely caused by ascending migration of female pinworms through the genital tract, with a prior pinworm infection acting as a predisposing factor. Her right ovarian serous cystadenoma was completely resolved following surgical resection. No active pinworm infection was detected during admission, as evidenced by negative adhesive tape tests and absence of anal itching; therefore, no additional anti-parasitic treatment was administered. Telephone follow-up over 6 months showed no recurrence of symptoms, and her overall health remained stable.

The initial misdiagnosis highlights two critical gaps in clinical practice across China. First, there is limited proficiency among healthcare personnel at all levels in distinguishing parasitic morphologies and interpreting paraffin-embedded pathological sections. Second, insufficient communication between clinicians and pathologists contributed to the diagnostic error. Ectopic enterobiasis should be considered in the differential diagnosis for female patients presenting with lower abdominal pain or pelvic masses, particularly in those with a history of pinworm infection.

To address these gaps and improve patient outcomes, we recommend three key interventions. First, provide targeted standardized training for primary healthcare workers, pathologists, and medical staff in non-endemic areas on the morphology of common parasites, including *E. vermicularis* and schistosomes, and the interpretation of histopathological sections. Second, strengthen collaboration between clinicians and pathologists, ensuring timely communication of epidemiological context, such as travel history to endemic areas, and relevant clinical history. Third, implement comprehensive management strategies, including synchronized family-unit treatment, thorough environmental cleaning, and two-course medication to interrupt transmission. Accurate diagnosis relies on a comprehensive approach integrating pathological examination as the cornerstone, clinical-pathological collaboration, and meticulous epidemiological assessment. Such an approach is critical for avoiding misdiagnosis, unnecessary interventions, and improving care for both typical and ectopic pinworm infections.

## Supplementary Information

Additional file 1: Supplementary Figure S1. Pathological section of the patient's greater omentum. Hematoxylin and eosin staining show a structure initially misinterpreted as a schistosome egg, which is in fact a cross-section of an adult female *Enterobius vermicularis*.

Additional file 2: Supplementary Figure S2. Pathological section of the greater omentum. Hematoxylin and eosin staining; arrows indicate structures initially misidentified as schistosome eggs, which are actually sections of pinworm eggs within the uterus of an adult *Enterobius vermicularis*.

## Data Availability

No datasets were generated or analysed during the current study.

## References

[1] Wendt S, Trawinski H, Schubert S, Rodloff AC, Mössner J, Lübbert C. The diagnosis and treatment of pinworm infection. Dtsch Arztebl Int. 2019;116(13):213–9. 31064642 10.3238/arztebl.2019.0213 PMC6522669

[2] Gunaratna GP, Dempsey S, Ho C, Britton PN. Diagnosis of *Enterobius vermicularis* infestations. J Paediatr Child Health. 2020;56(12):1994. 33351240 10.1111/jpc.15188

[3] Huang J, Zhu H, Zhou C, Zhu T, Zhang M, Chen Y, et al. Epidemiological profile and spatial patterns of enterobiasis in children aged 3–9 years in China from 2016 to 2020. Trop Med Infect Dis. 2022;8(1):25. 36668932 10.3390/tropicalmed8010025 PMC9866525

[4] Gaurav V, Kumar LP, Goyal D, Juyal D, Dev T. Pruritic vulvovaginitis in a young girl: a case of vulvovaginal enterobiasis. Pediatr Dermatol. 2025;42(3):663–5. 39731459 10.1111/pde.15834

[5] Pinto HA, Geiger SM, Melo AL, Mati VLT. Enterobiasis as a neglected worldwide disease: a call to action. Rev Soc Bras Med Trop. 2024;57:e011022024. 39476079 10.1590/0037-8682-0290-2024 PMC11524591

[6] Miao F, Zhang BG, Wang YB, Bu XQ, Zhang DB, Kong XL, et al. Survey of intestinal parasitic infections in rural residents of plain areas in Shandong Province. Zhongguo Xue Xi Chong Bing Fang Zhi Za Zhi. 2015;27(4):395–8 (in Chinese). 26767263

[7] Di Cicco M, Bertolucci G, Gerini C, Bruschi F, Peroni DG. Eosinophilia and potential antibody cross-reactivity between parasites in a child with pinworm and immune dysregulation: a case report. BMC Pediatr. 2023;23:200. 37101158 10.1186/s12887-023-04006-0 PMC10134642

[8] Takač I, Kavalar R, Lovrec MR, Lovrec VG. Concomitant ectopic *Enterobius vermicularis* infection in uterine cervical cancer. BMC Womens Health. 2024;24(1):265. 38678281 10.1186/s12905-024-03073-4 PMC11055370

[9] Racková J, Koutníková H, Kolářová Z, Neumannová H, Zikán M. A large adnexal tumor caused by *Enterobius vermicularis* mimicking malignancy. Helminthologia. 2022;59(4):373–6. 36875679 10.2478/helm-2022-0037 PMC9979071

[10] Roodbarani S, Ghasemikhah R. Diagnostic challenges, atypical presentations, and therapeutic implications in ectopic *Enterobius vermicularis* infections: a global systematic review. Diagn Microbiol Infect Dis. 2026;114(2):117136. 41086686 10.1016/j.diagmicrobio.2025.117136

[11] Rizvi G, Rawat V, Pandey HS, Kumar M. Acute abdomen: an uncommon presentation of a common intestinal nematode. Trop Parasitol. 2015;5(2):123–6. 26629456 10.4103/2229-5070.162526 PMC4557152

[12] Jawabreh I, Amro A, Azmi K, Batran H, Abdeen Z, Hamarsheh O. *Enterobius vermicularis* (pinworm) infestation mimicking acute appendicitis in two children from Palestine: a case report. J Med Case Rep. 2024;18(1):445. 39313844 10.1186/s13256-024-04785-9 PMC11421115

[13] Riedel J, Halm U, Prause C, Vollrath F, Friedrich N, Weidel A, et al. Multilocular hepatic masses due to *Enterobius vermicularis*. Inn Med (Heidelb). 2023;64(5):490–3. 36732426 10.1007/s00108-023-01479-0

[14] Serpytis M, Seinin D. Fatal case of ectopic enterobiasis: *Enterobius vermicularis* in the kidneys. Scand J Urol Nephrol. 2012;46(1):70–2. 21879805 10.3109/00365599.2011.609834

[15] Zahariou A, Karamouti M, Papaioannou P. *Enterobius vermicularis* in the male urinary tract: a case report. J Med Case Rep. 2007;1:137. 18001478 10.1186/1752-1947-1-137 PMC2194705

[16] Zafar S, Tariq MU, Ahmed Z. Ectopic *Enterobius vermicularis* infestation; an extremely rare cause of mesenteric lymphadenopathy mimicking tuberculous lymphadenitis. J Ayub Med Coll Abbottabad. 2018;30(1):124–6. 29504348

[17] O’Brien S, Ahmed S, Hayes B, O’Riordain M. Large-bowel obstruction secondary to *Enterobius vermicularis* pseudotumour. BMJ Case Rep. 2022;15(11):e252676. 36446475 10.1136/bcr-2022-252676 PMC9710356

[18] Al-Shouli ST, Barry M, Binkhamis K, AlHogail N, Alafaleq NO, Dufailu OA, et al. Fatal case of a child harboring *Enterobius vermicularis*. Healthcare. 2023;11(6):917. 36981574 10.3390/healthcare11060917 PMC10048790

[19] Terai J, Osada A, Tanaka M, Mitsuo A. Hazy omentum as a feature of paragonimiasis. Intern Med. 2024;63(10):1521–2. 37813612 10.2169/internalmedicine.2543-23 PMC11157309

[20] Kılıç S, Ekinci S, Orhan D, Senocak ME. *Enterobius granuloma*: an unusual cause of omental mass in an 11-year-old girl. Turk J Pediatr. 2014;56(2):189–91. 24911856

[21] Shen X, Zhang W, Wang PJ. CT manifestation of abdomen and its pathology of patients with chronic schistosomiasis. Zhongguo Xue Xi Chong Bing Fang Zhi Za Zhi. 2012;24(2):200–2 (In Chinese). 22799169

[22] Shim SS, Kim Y, Lee JK, Lee JH, Song DE. Pleuropulmonary and abdominal paragonimiasis: CT and ultrasound findings. Br J Radiol. 2012;85(1012):403–10. 22457403 10.1259/bjr/30366021 PMC3486672

[23] Ponniah K, Yong STL, Jayasooriya D. *Enterobius vermicularis* infestation: a rare cause of appendicitis. Cureus. 2025;17(2):e78924. 40091930 10.7759/cureus.78924 PMC11909491

[24] Heininger A, Densmore JC. *Enterobius vermicularis*: coming soon to an appendix near you? Cureus. 2025;17(5):e84894. 40575225 10.7759/cureus.84894 PMC12199720

[25] Murata K, Hasegawa H, Nakano T, Noda A, Yanai T. Fatal infection with human pinworm, *Enterobius vermicularis*, in a captive chimpanzee. J Med Primatol. 2002;31(2):104–8. 12110054 10.1034/j.1600-0684.2002.01017.x

[26] Raju K, Verappa S, Venkataramappa SM. *Enterobius vermicularis* infestation masquerading as cervical carcinoma: a cytological diagnosis. J Nat Sci Biol Med. 2015;6(2):476–9. 26283859 10.4103/0976-9668.160047 PMC4518439

