# Supplementary figures and images for: Rare Enterobius vermicularis infection of the greater omentum misdiagnosed as schistosomiasis: a case report
Source: Infect Dis Poverty. 2026 Mar 3;15:28. doi: 10.1186/s40249-026-01429-6 (PMC12954977; doi:10.1186/s40249-026-01429-6)

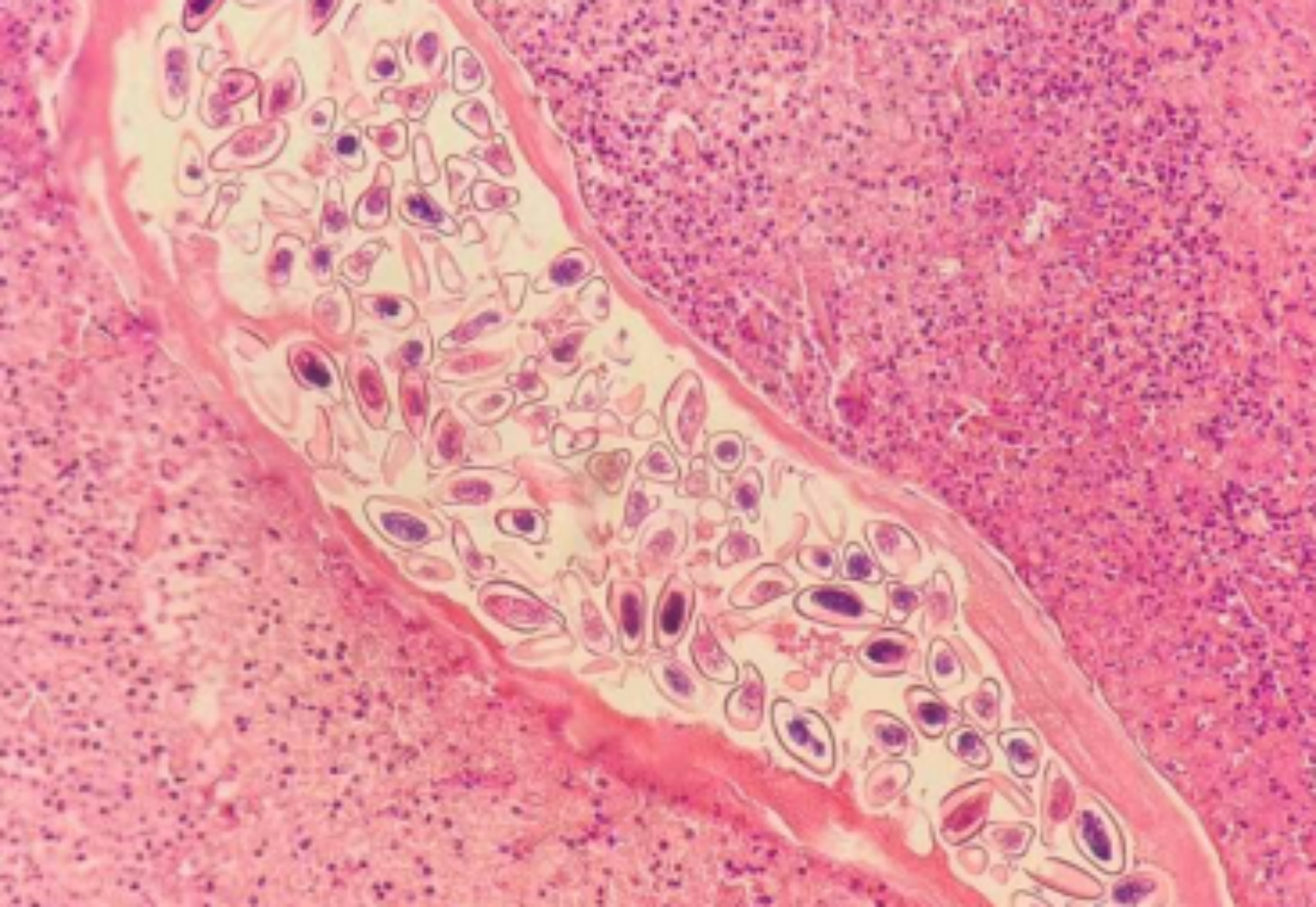

Supplement: Supplementary file 1 — Additional file 1: Supplementary Figure S1. Pathological section of the patient's greater omentum. Hematoxylin and eosin staining show a structure initially misinterpreted as a schistosome egg, which is in fact a cross-section of an adult female Enterobius vermicularis. [file 40249_2026_1429_MOESM1_ESM.tiff]

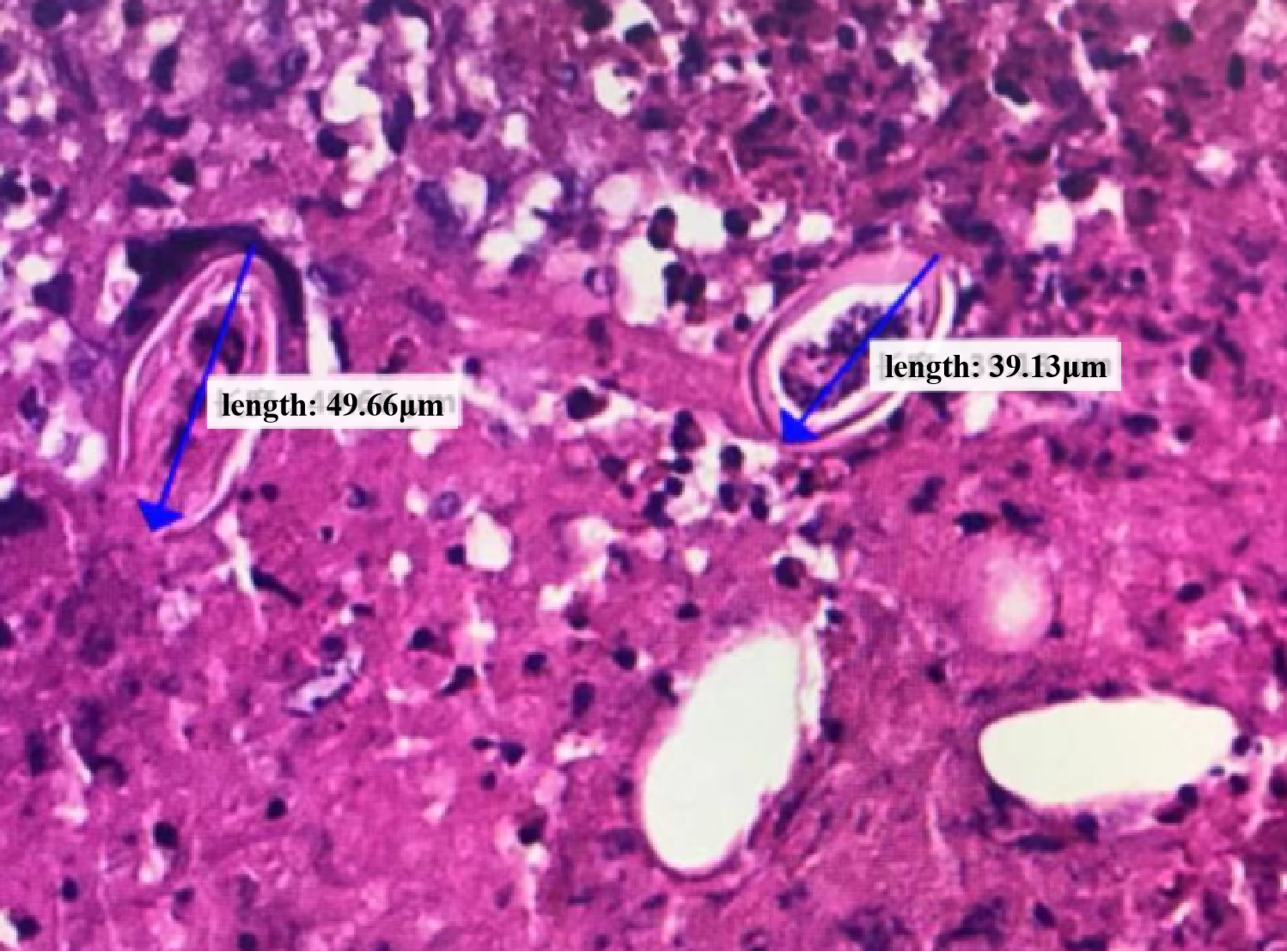

Supplement: Supplementary file 2 — Additional file 2: Supplementary Figure S2. Pathological section of the greater omentum. Hematoxylin and eosin staining; arrows indicate structures initially misidentified as schistosome eggs, which are actually sections of pinworm eggs within the uterus of an adult Enterobius vermicularis. [file 40249_2026_1429_MOESM2_ESM.tiff]
